# Supplementary material for: A Population-Based Analysis of the Cancer Incidence in Individuals under 50 in a Northern Italian Province: Focusing on Regional Disparities and Public Health Implications
Source: Int J Environ Res Public Health. 2024 Oct 8;21(10):1333. doi: 10.3390/ijerph21101333 (PMC11508065; doi:10.3390/ijerph21101333)
Supplement: Supplementary file 1 [file ijerph-21-01333-s001.zip › ijerph-3164558-supplementary.pdf]

Figure S1. Reggio Emilia Cancer Registry. Years 1996-2021. Incidence trends *in over 50 population* for all cancers (A), and for breast (B), testicular (C), lung (D), colorectal (E), thyroid (F) and melanoma (G).

Fig. S1A

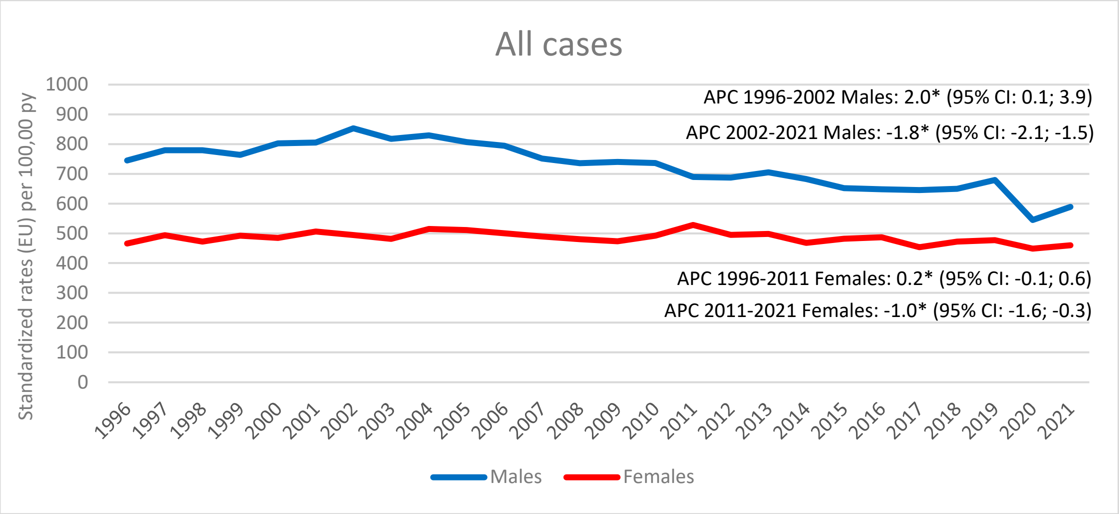

Fig. S1B

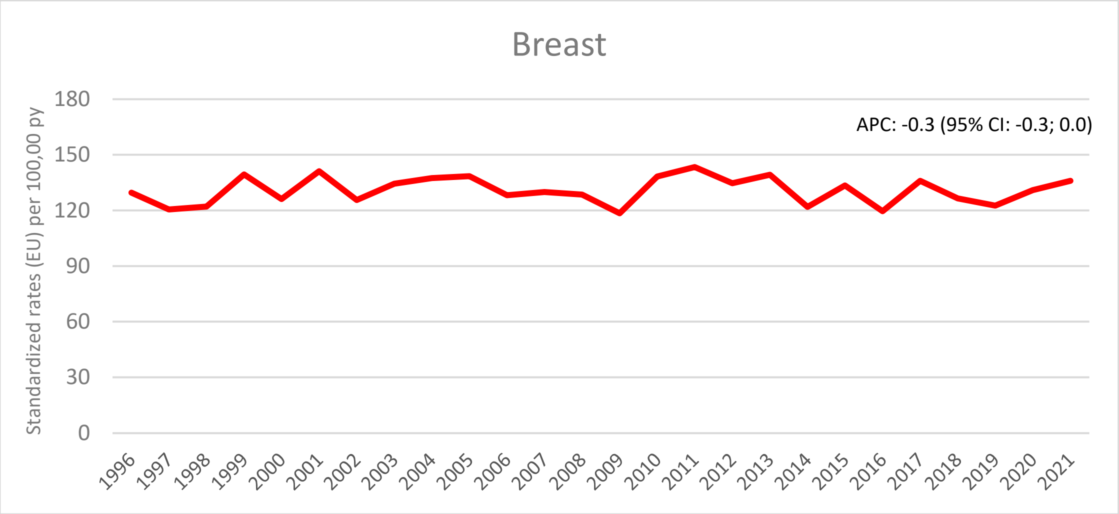

Fig. S1C

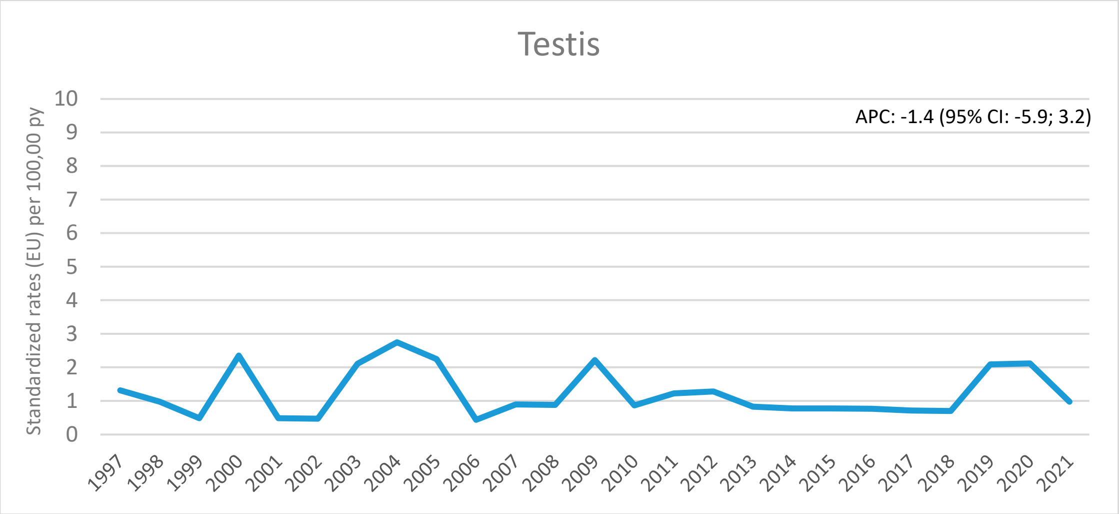

Fig. S1D

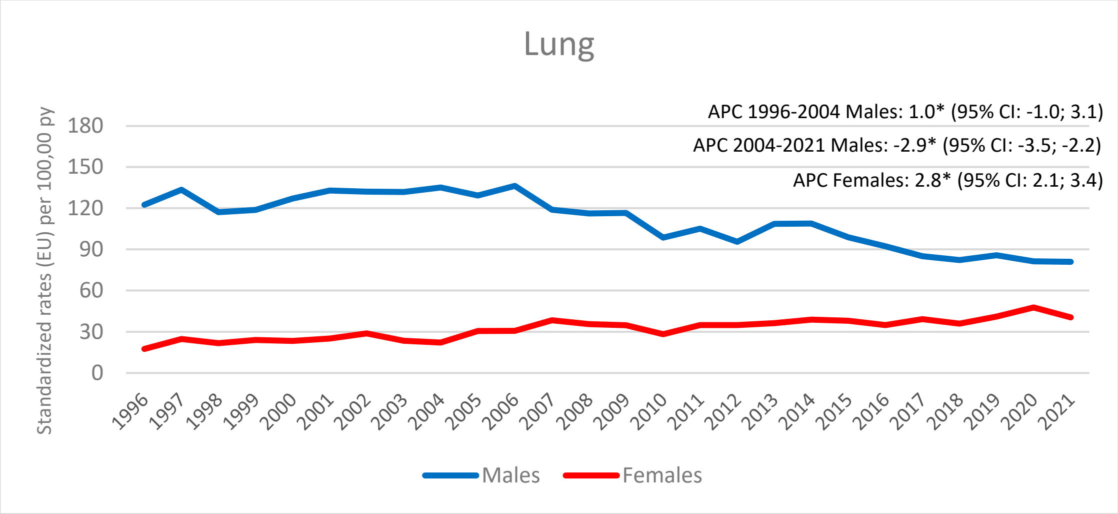

Fig. S1E

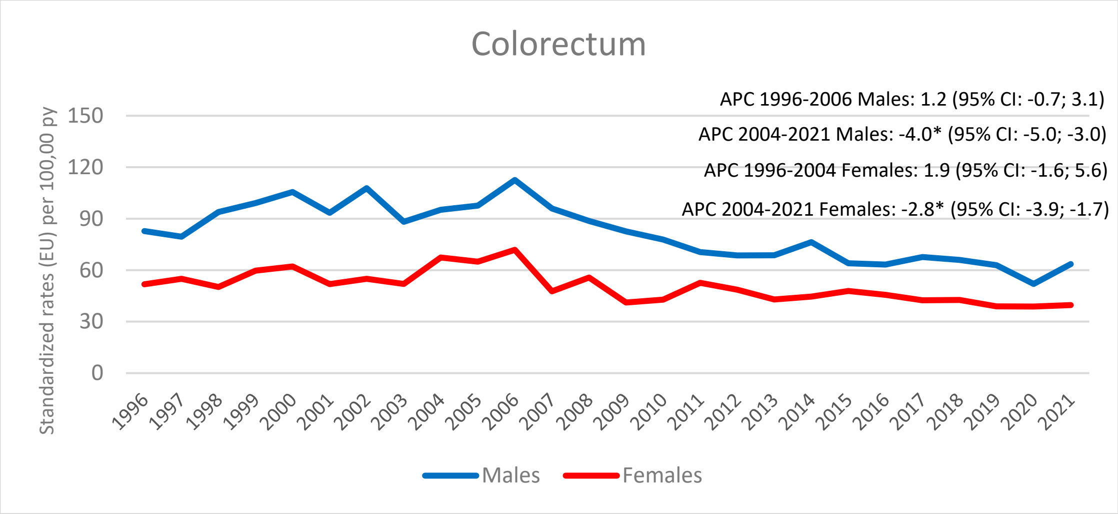

Fig. S1F

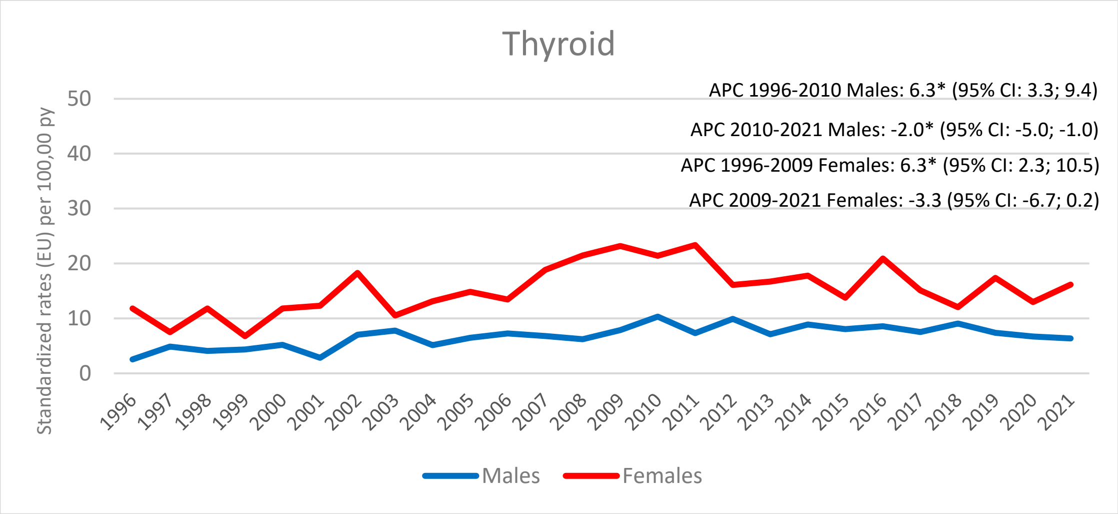

Fig. S1G

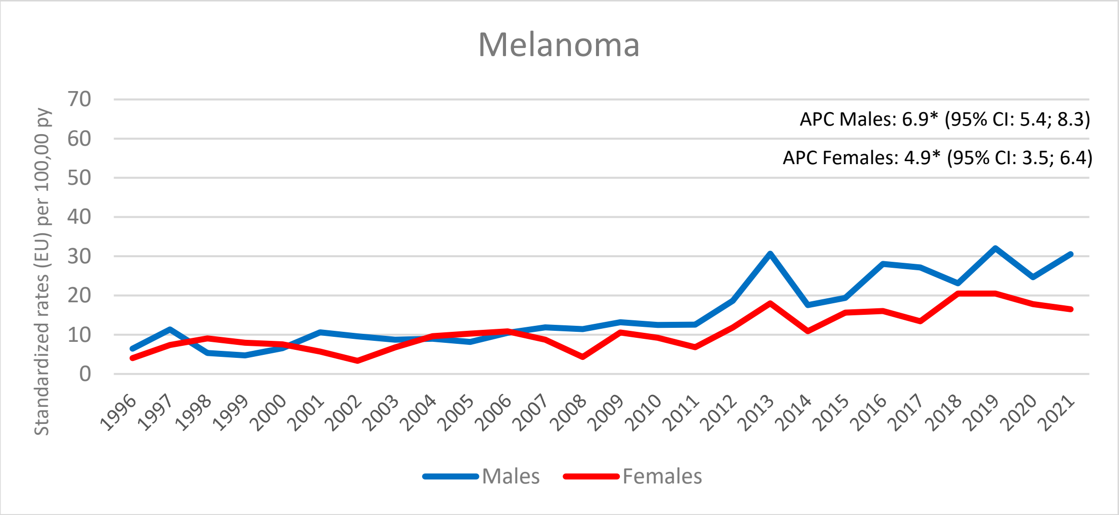

Melanoma
Standardized rates (EU) per 100,00 py
70
60
50
40
30
20
10
0
APC Males: 6.9* (95% CI: 5.4; 8.3)
APC Females: 4.9* (95% CI: 3.5; 6.4)
1996 1997 1998 1999 2000 2001 2002 2003 2004 2005 2006 2007 2008 2009 2010 2011 2012 2013 2014 2015 2016 2017 2018 2019 2020 2021
Males
Females
